# Highly Efficient ITO-Free Quantum-Dot Light Emitting Diodes via Solution-Processed PEDOT:PSS Semitransparent Electrode

**DOI:** 10.3390/ma16114053

**Published:** 2023-05-29

**Authors:** Jin Hyun Ma, Min Gye Kim, Jun Hyung Jeong, Min Ho Park, Hyoun Ji Ha, Seong Jae Kang, Seong Jun Kang

**Affiliations:** 1 Department of Advanced Materials Engineering for Information and Electronics, Kyung Hee University, Yongin 17104, Republic of Koreamgee96@khu.ac.kr (M.G.K.); 2016101120@khu.ac.kr (J.H.J.); triolight@khu.ac.kr (M.H.P.); jrhjhmpapa@gmail.com (H.J.H.); andrew970728@khu.ac.kr (S.J.K.); 2 Integrated Education Program for Frontier Materials (BK21 Four), Kyung Hee University, Yongin 17104, Republic of Korea

**Keywords:** quantum-dots (QDs), light-emitting diodes, ITO-free, PEDOT:PSS electrodes, energy-level alignment, high efficiency

## Abstract

We present a study on the potential use of sulfuric acid-treated poly(3,4-ethylenedioxythiophene):poly(styrene sulfonate) (PEDOT:PSS) as a viable alternative to indium tin oxide (ITO) electrodes in quantum dot light-emitting diodes (QLEDs). ITO, despite its high conductivity and transparency, is known for its disadvantages of being brittle, fragile, and expensive. Furthermore, due to the high hole injection barrier of quantum dots, the need for electrodes with a higher work function is becoming more significant. In this report, we present solution-processed, sulfuric acid-treated PEDOT:PSS electrodes for highly efficient QLEDs. The high work function of the PEDOT:PSS electrodes improved the performance of the QLEDs by facilitating hole injection. We demonstrated the recrystallization and conductivity enhancement of PEDOT:PSS upon sulfuric acid treatment using X-ray photoelectron spectroscopy and Hall measurement. Ultraviolet photoelectron spectroscopy (UPS) analysis of QLEDs showed that sulfuric acid-treated PEDOT:PSS exhibited a higher work function than ITO. The maximum current efficiency and external quantum efficiency based on the PEDOT:PSS electrode QLEDs were measured as 46.53 cd/A and 11.01%, which were three times greater than ITO electrode QLEDs. These findings suggest that PEDOT:PSS can serve as a promising replacement for ITO electrodes in the development of ITO-free QLED devices.

## 1. Introduction

Indium tin oxide (ITO) is a commonly used transparent electrode due to its high transparency, conductivity, and chemical stability [1]. For these reasons, ITO electrodes are widely used in fields such as light-emitting diodes, solar cells, sensors, and thin-film transistors [2,3,4,5]. However, ITO has several disadvantages, such as its brittleness, ease of scratching, and the necessity of a vacuum process for its fabrication. Especially, the price of ITO is high due to its scarcity and the high demand for indium [6]. To address these limitations, researchers are exploring alternative materials such as graphene, carbon nanotubes, silver nanowires, and conductive polymers (Table S1) [7,8,9,10,11,12,13,14,15]. Among them, poly(3,4-ethylenedioxythiophene):poly(styrenesulfonate) (PEDOT:PSS) is a promising material that can replace ITO due to its low cost, high conductivity, transparency, and ability for solution processing.

Several previous studies have attempted to increase the conductivity of PEDOT:PSS using methods such as post-treatment with solvents such as DMSO, acid treatment, adding additive, and doping with ionic material [16,17,18,19,20]. Kim et al., reported a method of significantly increasing the conductivity of PEDOT:PSS CLEVIOSTM PH1000 (PH1000) by dipping in sulfuric acid [17]. Despite the high conductivity of PEDOT:PSS, which makes it a promising alternative to ITO, few studies have investigated the impact of PEDOT:PSS as an electrode on the electrical properties of devices [21,22]. The higher work function value of PEDOT:PSS compared to ITO represents a clear advantage, particularly for hole injection applications [23]. The higher work function of PEDOT:PSS allows for more efficient injection of holes, thereby enabling improved device performance. This attribute makes PEDOT:PSS electrodes suitable for utilization in various fields where efficient hole injection is crucial. Therefore, while the enhanced conductivity of PEDOT:PSS shows potential as a replacement for ITO electrodes, its higher work function value provides additional benefits that are highly valuable in applications.

In terms of energy-level alignment, quantum-dot light-emitting diodes (QLEDs) face challenges related to the charge imbalance between holes and electrons [24]. The effective mass difference between electrons and holes, coupled with a high hole injection barrier of quantum dots (QDs), can lead to a charge imbalance that negatively impacts the luminance value, current efficiency (CE), and external quantum efficiency (EQE) irrespective of the QD’s bandgap [25,26,27]. To address this problem, researchers have reported studies on reducing the electron injection speed by forming an electron transport layer in a double layer or further manufacturing a hole transport layer to facilitate hole injection [28,29,30]. These studies have typically focused on solving charge imbalance using ITO electrodes. Rather than facilitating additional layers, which complicate the device structure and fabrication process, it has been suggested that using electrodes with a high work function can be a more efficient approach to address charge imbalance and produce high-efficiency QLEDs [31].

Here, we introduce a study on improving the CE and EQE of green QLEDs by using solution-processed, sulfuric acid-treated PH1000 (H-PH1000) as an anode. Although H-PH1000 electrodes have lower transparency properties than ITO, their higher work function facilitates injection by lowering the barrier between the electrodes and the hole injection layer. In the optimized device using a three-layer H-PH1000 electrode, the maximum luminance, CE, and EQE values are 46,663 cd/m^2^, 46.53 cd/A, and 11.01%, respectively. The CE and EQE values of the QLEDs using the H-PH1000 electrode were three times higher than those of the ITO QLEDs. Furthermore, we investigated the electroluminescence characteristics and utilized hole-only devices to gain insight into the behavior of alternative electrodes beyond ITO. Our observations revealed that band alignment plays a crucial role in the low-voltage region, while the electrode’s resistance becomes more prominent at higher voltages. These findings further support the notion that the improved performance of QLEDs can be attributed to the high work function of the alternative electrode. Overall, our study demonstrates that the use of solution-processed, sulfuric acid-treated H-PH1000 electrodes offers a viable approach to enhance the efficiency of QLEDs, surpassing the efficiency of traditional ITO-based QLEDs.

## 2. Experimental Section

### 2.1. H-PH1000 Electrodes Fabrication

The bare glass substrates (1.5 cm × 1.5 cm, AMG, Uiwang, Republic of Korea) were prepared by cleaning in ultrasonic baths (DH.WUC.A03H, DAIHAN-scientific) of DI water, acetone, and IPA for 15 min, respectively. After drying the substrates with N_2_ gas blowing, they were treated with UV-ozone (UVC-150, Omniscience) for 15 min to enhance surface hydrophilicity and remove organic residue. A solution of PH1000 (Heraeus, Germany) was then spin-coated onto the substrate at 3000 rpm for 30 s and pre-annealed at 100 °C for 5 min. This spin-coating process was repeated to produce 1-, 2-, and 3-layer PH1000 films, which were then post-annealed at 150 °C for 15 min. To increase the conductivity of the PH1000, the sequentially formed PH1000 films were dipped in 98% sulfuric acid (Duksan General Science, Seoul, Republic of Korea) for 10 min, followed by rinsing with DI water to remove residual acid. The H-PH1000 substrates were then annealed at 150 °C for 5 min. The substrates were scribed to a size of approximately 1.5 cm × 0.5 cm using IPA and isolated using Kapton tape and polyethylene naphthalate (PEN) on the non-removable side to prevent contamination in the subsequent spin-coating process. The fabrication details for the dipping and isolation processes are provided in Figure S1.

### 2.2. Device Fabrication

On the prepared H-PH1000 electrodes, a solution of PEDOT:PSS CLEVIOS P AI4083 (AI4083) (Heraeus, Germany) was spin-coated at 3000 rpm for 30 s and annealed at 150 °C for 15 min. Then, a 1 wt% solution of poly[(9,9-dioctylfluorenyl-2,7-diyl)-co-(4,4′-(N-(4-sec-butylphenyl)diphenylamine)] (TFB) (Lumtec, LT-N148), dispersed in p-xylene (Sigma-Aldrich), was spin-coated at 3000 rpm for 30 s and annealed at 180 °C for 30 min. The CdSe/ZnS QDs (Uniam, 20 mg/mL) solution was spin-coated on the TFB layer at 2000 rpm for 30 s and annealed at 90 °C for 10 min. The ZnO nanoparticles (Avantama, N-10) were spin-coated on the QDs thin film at 2000 rpm for 60 s and then annealed at 60 °C for 10 min. Finally, an aluminum (Al) cathode was deposited for 130 nm using a thermal evaporator at a rate of 3 Å/s with a shadow metal mask.

### 2.3. Characterization

The surface morphologies of the films were observed using an atomic force microscopy (AFM) (S.I.S-GmbH, Berlin, Germany), and the transmittance was measured using a UV-visible spectroscopy (Cary 100, Agilent, Santa Clara, CA, USA). The sheet resistance and hole mobility were characterized via Hall measurement (HL 5500 PC, Nanometrics, Hillsboro, OR, USA). The contact angle (CA) was measured using a Phoenix 300 (SEO, Suwon, Republic of Korea). The cross-sectional image of the QLED was investigated using a high-resolution transmission electron microscope (HR-TEM) (JEM-2100F, JEOL, Seoul, Republic of Korea). To determine the chemical state and the energy-level alignment at the interface, measurements were conducted using X-ray photoelectron spectroscopy (XPS) (K-Alpha, Thermo Electron, Waltham, MA, USA) and ultraviolet photoelectron spectroscopy (UPS) (NEXA, Thermo Fisher Scientific, Waltham, MA, USA) with Al Kα (1486.8 eV) and He I (21.22 eV) light sources, respectively. The electroluminescence (EL) properties and Commission International l’eclaireage 1931 (CIE 1931) (x,y) coordinates were evaluated via the OLED I-V-L measurement system (M-6100, McScience, Suwon, Republic of Korea).

## 3. Results and Discussion

### 3.1. Characterization of H-PH1000 Electrodes

**Figure 1:**
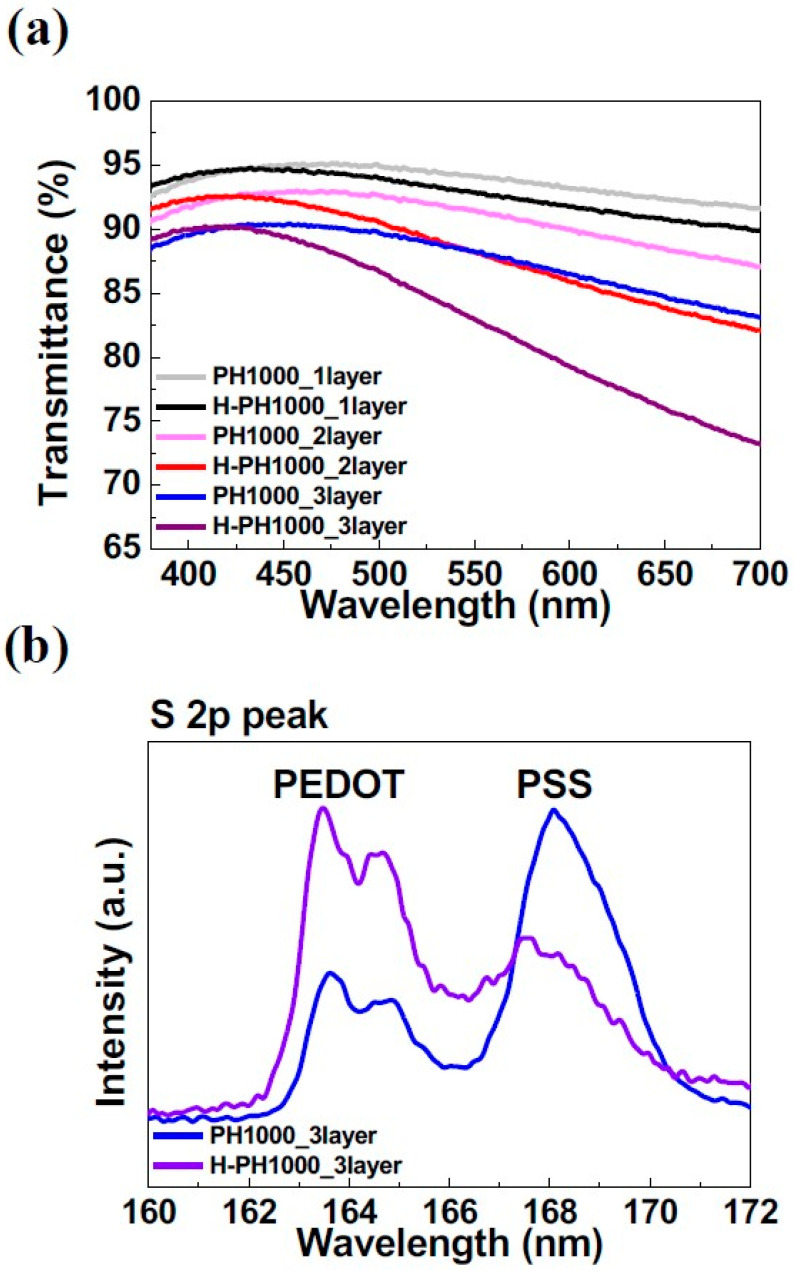
(**a**) Transmittance spectra of the PH1000 and H-PH1000 films with 1-, 2- and 3-layers. (**b**) Sulfur 2p XPS spectra of 3-layer PH1000 and H-PH1000 films.

The optical characteristics of the PH1000 and H-PH1000 films are shown in Figure 1a. The transmittance of one-, two-, and three-layer PH1000s were 94.14%, 91.40%, and 88.22% at 550 nm, as measured by UV-visible spectroscopy. Sarker et al. reported an increase in conductivity when hydroiodic acid treatment was applied to multi-layered PH1000 [18]. However, since the Cas of PH1000 droplets on one-, two-, and three-layer PH1000 films were 41°, 49°, and 57°, stacking more than three layers of PH1000 was difficult, as shown in Figure S2. When treated with sulfuric acid, the transmittance values of one-, two-, and three-layer H-PH1000s were 92.82%, 88.19%, and 82.94%, and it was observed that the transmittance values decreased. This is because, when treated with sulfuric acid, recrystallized PH1000 becomes metallic and shows a higher reflectance [17,32]. It is worth noting that the three-layer H-PH1000 film still maintained a semi-transparent property despite the decrease in transmittance.

XPS analysis was performed to examine the chemical composition of the three-layer H-PH1000. The XPS spectra of the sulfur 2p (S 2p) peaks are shown in Figure 1b. At three-layer PH1000, the XPS spectra of the PEDOT core level exhibited a peak at the binding energy around 163–165 eV, and the PSS core level showed a PSS peak around 167–169 eV [33]. As the three-layer PH1000 was dipped in sulfuric acid, the PEDOT peak significantly increased, and the PSS peak decreased. In particular, the PSS peak shifted to a lower binding energy, which indicates the conversion of hydrogen bonds for the chains of poly(styrenesulfonic acid) (PSSH) to PSS through sulfuric acid treatment. The decrease in the peak of PSS is due to the removal of the PSSH region in the process of recrystallization with acid treatment [34]. This PEDOT-rich H-PH1000 film is known to increase conductivity [35].

**Figure 2:**
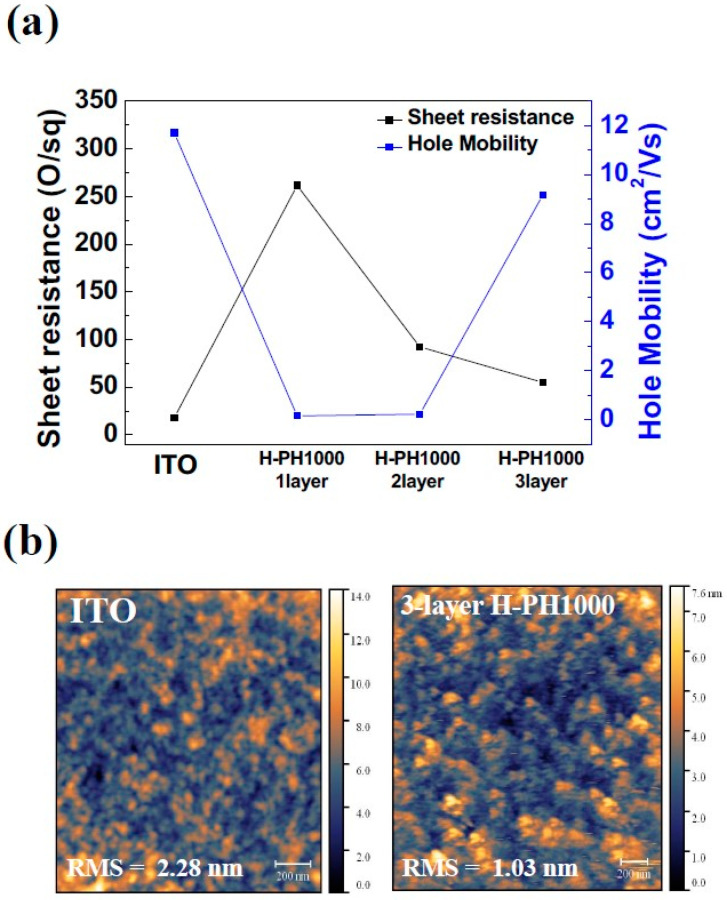
(**a**) Comparison of electrical characteristics between H-PH1000 and ITO electrodes. (**b**) AFM images of 3-layer H-PH1000 and ITO films.

To evaluate the potential of H-PH1000 as electrodes for QLEDs, its electrical properties were measured using Hall measurement. The sheet resistance and hole mobility of one-, two-, and three-layer H-PH1000 and ITO are shown in Figure 2a. The sheet resistance values for ITO and one-, two-, and three-layer H-PH1000 were 18 Ω/square, 261.4 Ω/square, 92.11 Ω/square, and 55.06 Ω/square. Figure S3 shows the distribution of the sheet resistance of the H-PH1000 layers. According to the increase in the number of H-PH1000, the sheet resistance was decreased. The hole mobility values for ITO and one-, two-, and three-layer H-PH1000 were 11.7 cm^2^/Vs, 0.162 cm^2^/Vs, 0.227 cm^2^/Vs, and 9.17 cm^2^/Vs, respectively. The increased mobility in three-layer H-PH1000 compared to one- and two-layer H-PH1000 is attributed to the thickness-dependent characteristics of the PEDOT:PSS films. In the study by Kim et al., they classified multilayer PEDOT:PSS films based on their thickness after treatment with sulfuric acid and DMSO. It was found that thinner films exhibited a relatively amorphous state, while thicker films showed decreased surface roughness and increased mobility due to crystallization [33]. Notably, the hole mobility value of three-layer H-PH1000 was not significantly lower than ITO, despite the higher sheet resistance value. The reason for this could be attributed to the difference in surface roughness. The surface roughness values for ITO and three-layer H-PH1000 were 2.28 nm and 1.03 nm (Figure 2b). The hole mobility value can be influenced by the surface roughness, and in comparison to ITO, three-layer H-PH1000 exhibited a smoother surface roughness, which led to a relatively higher measured value [36]. These results suggest that optimal three-layer H-PH1000 can be a potential candidate for use as an electrode material in QLEDs, especially for applications where a smooth surface is required.

### 3.2. Performance of QLED with H-PH1000 Electrodes

**Figure 3:**
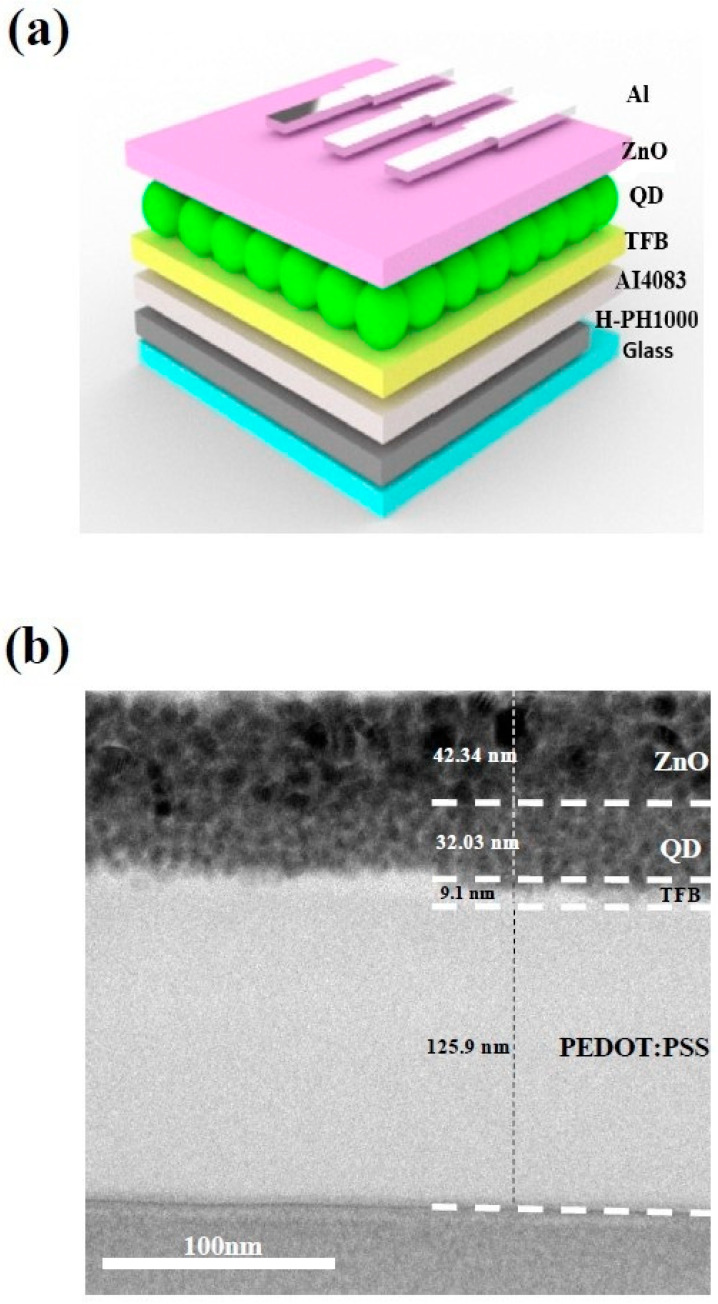
(**a**) Schematic structure of QLEDs with an H-PH1000 electrode. (**b**) Cross-sectional HR-TEM image of a 3-layer H-PH1000 QLED.

The QLED device structure using the three-layer H-PH1000 electrode is depicted in Figure 3a. The transmittance value of the electrode is crucial, as it is a bottom-emitting structure through the electrode side. To ensure the device’s optimal performance, it is important to achieve high-quality and defect-free layer stacking. To confirm the structural integrity of the QLED device, we conducted HR-TEM imaging, as shown in Figure 3b. The results indicated that the layers were stacked sequentially without any observable defects. The H-PH1000 layers were formed by spin-coating and dipping methods, resulting in a well-structured layer. However, distinguishing between the H-PH1000 electrode and AI4083, the hole injection layer, was challenging due to their identical material compositions. Overall, the structural integrity and transmittance value of the H-PH1000 electrode in the QLED device are critical for achieving high device performance.

**Figure 4:**
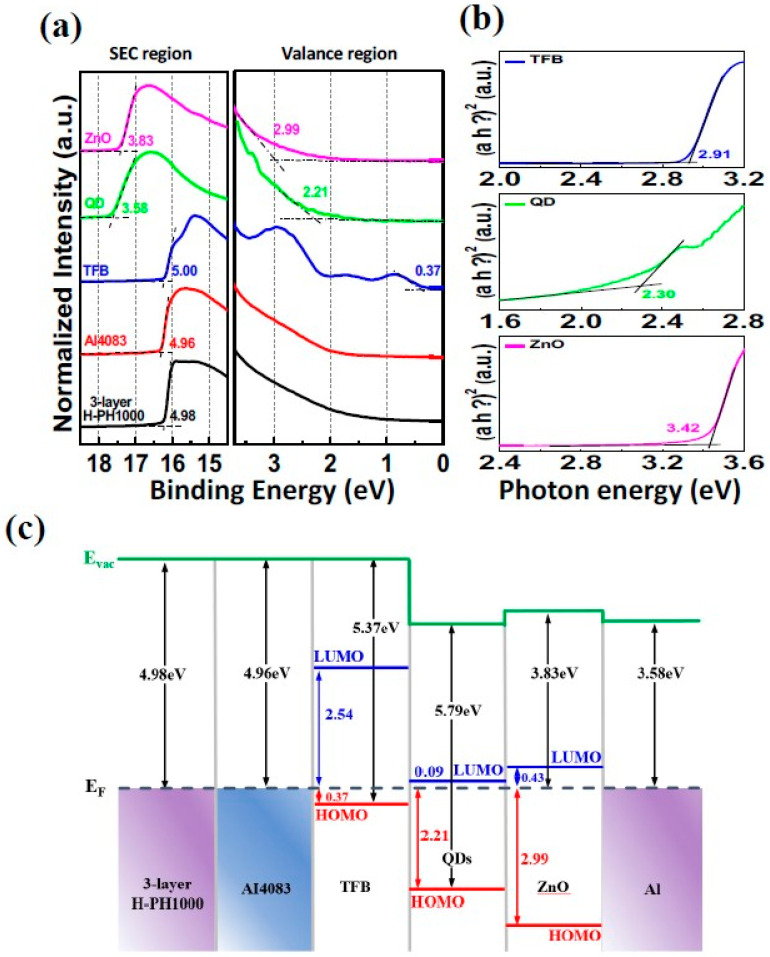
(**a**) SEC (left) and valence (right) region spectra of 3-layer H-PH1000, 3-layer H-PH1000/AI483, 3-layer H-PH1000/AI483/TFB, 3-layer H-PH1000/AI483/TFB/QDs, 3-layer H-PH1000/AI483/TFB/QDs/ZnO. (**b**) Optical bandgaps of TFB, QDs, and ZnO. (**c**) Interfacial energy-level diagram of QLED with H-PH1000 electrode.

UPS measurements were performed to determine the work function and energy level differences between the Fermi level and the occupied molecular orbital (HOMO) level in the various layers of the QLED device. The values for the secondary electron cutoff region (SEC region; left side) and valence region (right side) were obtained, as shown in Figure 4a.

The work function of the three-layer H-PH1000 electrode was determined to be 4.98 eV, by calculating the difference between the He I lamp (*hν* = 21.22 eV) and the SEC region. This value was found to be 0.44 eV higher than the work function of ITO measured in our previous study [37]. These results suggest that H-PH1000 may be more appropriate for hole injection, as the energy level differences between ITO and the hole injection layer can be problematic [38]. The energy level difference between the Fermi level and the HOMO level was determined using the valence region. Additionally, the optical band gap of each layer was measured through UV-visible spectroscopy using the Tauc plot method with the following equation:

(1) $$ahv=A{\left(hv-E_{g}\right)}^{n}$$

where α is the absorption coefficient, *hν* is the photon energy, A is a constant, E_g_ is the optical band gap, and *n* = 1/2 for a direct band gap semiconductor [39]. The optical bandgap values for the TFB, QDs, and ZnO layers were found to be 2.91 eV, 2.30 eV, and 3.42 eV, respectively (Figure 4b). Based on these results, the band alignment diagram of the QLED device is presented in Figure 4c. The QDs show that the difference between the Fermi level and the LUMO level is 0.09 eV, while the difference from the HOMO level is 2.21 eV. Through the analysis of band alignment, it becomes evident that the hole injection barrier is significantly greater than that of the electron for the carriers injected in order to facilitate light emission in QLEDs. The measured high work function of the H-PH1000 electrode, along with its favorable band alignment with quantum dot (QD) layers, is expected to enhance hole injection. This implies that the use of H-PH1000 electrodes can effectively reduce the hole injection barrier, enabling improved charge injection and ultimately enhancing the overall performance of QLEDs.

**Figure 5:**
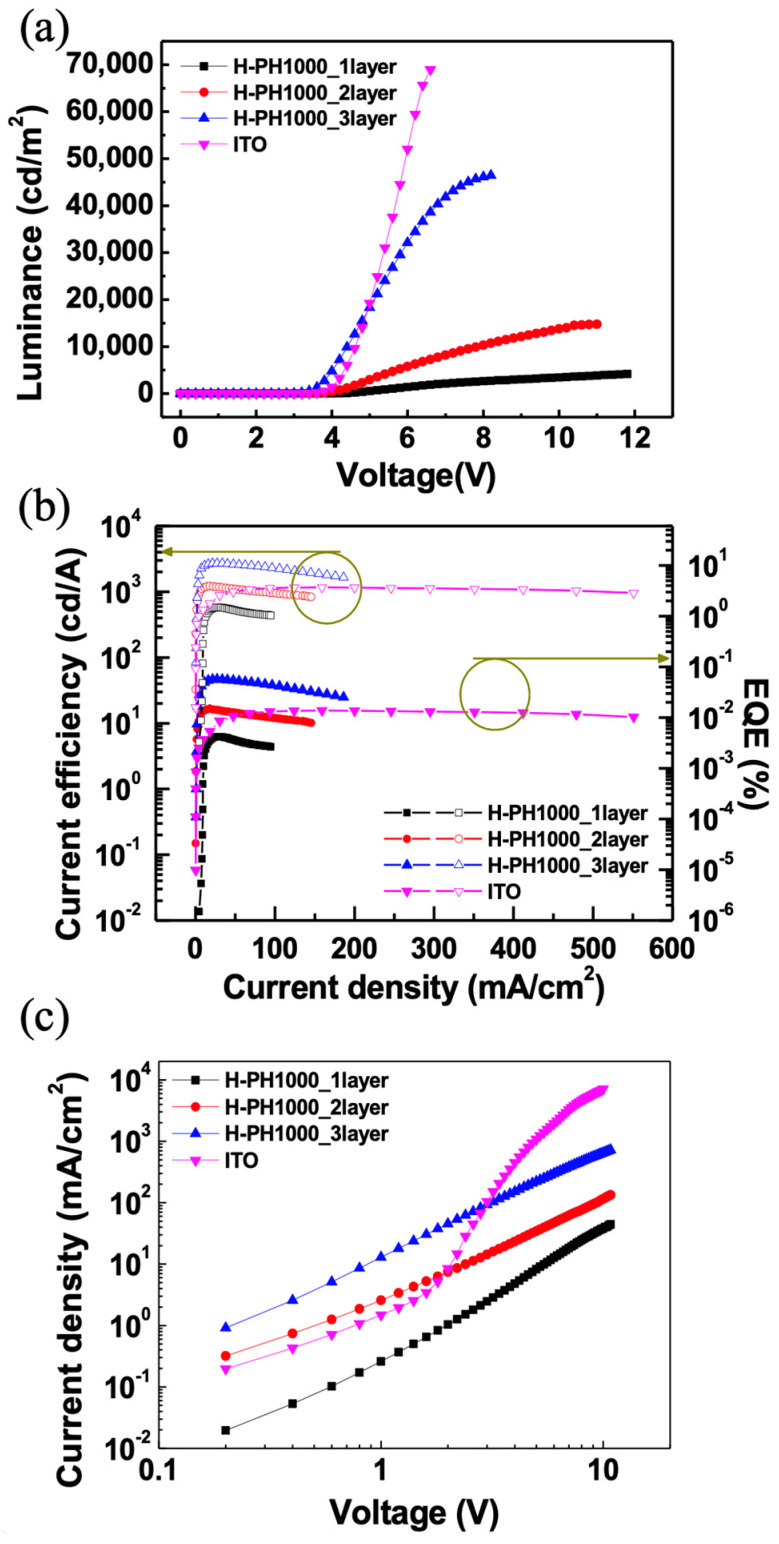
(**a**) Voltage−luminance characteristics and (**b**) CE−EQE−J characteristics of QLEDs (**c**) J−V characteristics of HODs with H-PH1000 and ITO electrodes.

Figure 5a,b shows the EL characteristics of QLEDs with one-, two-, and three-layer H-PH1000 and ITO electrodes. The summarized EL characteristics with various electrodes are described in Table 1. The maximum luminances of the QLEDs with one-, two-, and three-layer H-PH1000 and ITO electrodes were 4287 cd/m^2^, 14,679 cd/m^2^, 46,663 cd/m^2^, and 68,977 cd/m^2^, respectively. Due to the higher sheet resistance and absorption generated by the semi-transparent H-PH1000 electrodes, the H-PH1000 QLEDs exhibited lower luminance values than the ITO QLEDs, as the overall current density decreased. However, the turn-on voltages, defined at 1 cd/m^2^, for QLEDs with three-layer H-PH1000 and ITO are 2.6 V and 3.0 V. Moreover, the maximum CE and EQE of three-layer H-PH1000 electrodes QLEDs were 37.01 cd/m^2^ and 11.01 %. As shown in Table S2, the electroluminescence values of three-layer H-PH100 QLED exhibit higher performance compared to QLEDs employing the previously explored ITO replacement electrodes [40,41,42]. The main factor contributing to the observed increase in efficiency is the band alignment resulting from the H-PH1000 electrode’s high work function. This band alignment plays a crucial role in facilitating efficient hole injection and reducing the turn-on voltage. By establishing a favorable energy-level alignment at the electrode interface, the H-PH1000 electrode promotes efficient charge injection and balanced carrier transport within the QLED device. As a result, higher efficiency values were achieved compared to QLEDs employing ITO electrodes. The operational lifetime of the QLED devices was evaluated, as shown in Figure S4. The measurements were conducted under ambient air conditions with a relative humidity of approximately 50% in the constant current mode. The half-lifetimes (T_50_) of the QLED devices using the three-layer H-PH1000 electrode and ITO electrode were measured to be 14.5 min and 27.2 min, respectively. The decrease in overall lifetime observed when using the H-PH1000 electrode was attributed to the susceptibility of PEDOT:PSS to humidity [43].

The CE and EQE of H-PH1000 QLEDs showed a decrease, with the three-layer H-PH1000 QLED experiencing a decrease in both CE and EQE to 53.6% and 53.14% of the maximum value, respectively. This decrease was not attributed to roll-off, which is characterized by a decrease in EQE to 90% of the maximum value in OLEDs when the luminance value is increased from 1000 cd/m^2^ to 10,000 cd/m^2^ [44,45]. The EQE of three-layer H-PH1000 QLED increased from 6.41% to 10.95% when the luminance values were increased from 1302 cd/m^2^ to 9880 cd/m^2^. If the efficiency decreases over a wide range of voltages in QLEDs, rather than at the beginning, it can still be considered for practical applications based on current density [46]. As the voltage increased, the electrode’s resistance became more important, determining the current density value, as shown in Figure S5. The reason for the decrease in EQE was assumed to be a result of a charge imbalance caused by inadequate injection of holes due to the high sheet resistance of the H-PH1000 electrodes at high voltages. This indicates the need for a careful balance between the sheet resistance and charge transfer properties for QLED performance. 

To confirm the EL performances, the hole-only devices (HODs) were fabricated with device structures of bare glass/one-, two-, and three-layer H-PH1000 and ITO/AI4083/TFB/Al. Figure 5c shows the current density (*J*–*V*) characteristics of the HODs. In the low voltage range, two- and three-layer H-PH1000 QLEDs exhibit higher current density values compared to ITO due to band alignment, despite having higher resistance. However, as the voltage increases, the electrode’s resistance becomes the dominant factor, leading to higher current flow. The relation between the electrodes’ sheet resistance and charge transfer properties for QLEDs is consistent with HODs.

**Figure 6:**
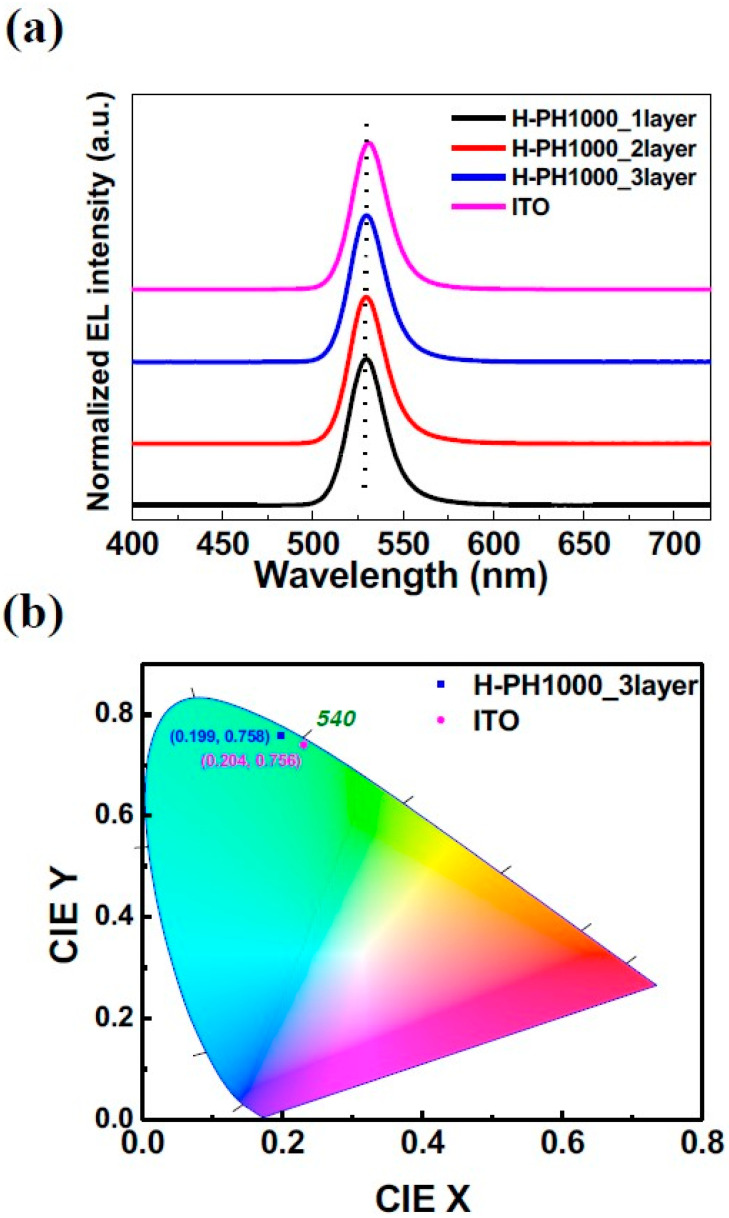
(**a**) EL spectra of QLEDs at respective maximum luminance. (**b**) CIE1931 coordinates of 3-layer H-PH1000 and ITO QLEDs at maximum luminance.

The EL spectra at the maximum luminance of each device are exhibited in Figure 6a. All QLEDs exhibit EL peaks with a high color purity and full width half maximum value of approximately 25 nm. As shown in Figure 6b, the CIE1931 (x,y) coordinates with three-layer H-PH1000 and ITO QLEDs were measured as (0.199, 0.758) and (0.204, 0.756). The EL peak of the ITO QLED appears to be blue-shifted compared to H-PH1000, possibly due to the Joule heat generated by the high current density [47]. The use of H-PH1000 as an electrode with a high work function offers a promising solution to improve the hole injection characteristics in QLEDs, ultimately resulting in the fabrication of high-efficiency QLEDs.

## 4. Conclusions

In this study, we fabricated high-efficiency QLEDs by utilizing sulfuric acid-treated PH1000 as electrodes. We investigated the effect of sulfuric acid treatment on PEDOT-rich film formation using XPS measurements, which confirmed the increase in conductivity after the treatment. UPS measurements were performed to investigate the work function of the layers and band alignment of the QLEDs. When H-PH1000 films were used as electrodes in QLEDs, the higher work function facilitated hole injection and improved the alignment properties, leading to enhanced device performance. To further investigate the effect of band alignment and sheet resistance of the electrodes, we conducted hole-only device measurements, which revealed that band alignment played a more significant role in the region of low voltage, while the sheet resistance of the electrodes acted dominantly in the region of voltage. We achieved a maximum luminance of 46,663 cd/m^2^, current efficiency of 46.53 cd/A, and external quantum efficiency of 11.01% when using three-layer H-PH1000 as an electrode. Our research findings provide evidence that the application of sulfuric acid-treated PH1000 holds immense potential for enhancing the hole injection characteristics in red or blue QLEDs. Moreover, these results indicate the potential of employing PEDOT:PSS electrodes in the OLED field to address the ongoing challenges associated with hole transport characteristics. By incorporating H-PH1000 electrodes across a range of optoelectronic devices, these issues are expected to be successfully mitigated, leading to enhanced overall performance [48,49,50].

## Figures and Tables

**Table 1:** Summarized EL characteristics of QLEDs with various electrodes.

Device	Max. L (cd/m^2^)	Turn on V (V)	Max. CE (cd/A)	Max. EQE (%)	CIE1931 (x,y)
H-PH1000_1layer	4287	3.4	6.22	1.48	(0.198, 0.758)
H-PH1000_2layer	14,679	3.2	10.67	3.93	(0.199, 0.759)
H-PH1000_3layer	46,663	2.6	46.53	11.01	(0.199, 0.758)
ITO	68,977	3.0	15.62	3.69	(0.204, 0.756)

## Data Availability

Data will be made available from the corresponding authors on reasonable request.

## Supplementary Materials

The following supporting information can be downloaded at: https://www.mdpi.com/article/10.3390/ma16114053/s1, Figure S1: Schematic illustration of H-PH1000 electrode fabrication, Figure S2: CA images with (a) 1-layer PH1000 (b) 2-layer PH1000 (c) 3-layer PH1000, Figure S3: Sheet resistance distribution of H-PH1000 layers, Figure S4: Operational lifetime measurements of 3-layer H-PH1000 and ITO QLEDs, Figure S5: Voltage-current density characteristics of QLEDs, Table S1: Characteristics comparison of ITO and alternative electrodes, Table S2: Summarized EL characteristics of QLEDs with alternatives to ITO electrodes.
