# Supplementary material for: Highly Efficient ITO-Free Quantum-Dot Light Emitting Diodes via Solution-Processed PEDOT:PSS Semitransparent Electrode
Source: Materials (Basel). 2023 May 29;16(11):4053. doi: 10.3390/ma16114053 (PMC10254849; doi:10.3390/ma16114053)
Supplement: Supplementary file 1 [file materials-16-04053-s001.zip › materials-2377308-supplementary.pdf]

Supporting Information

Highly efficient ITO-free quantum-dot light emitting diodes via solution processed PEDOT:PSS semitransparent electrode

Jin Hyun Ma[a,b], Min Gye Kim[a,b], Jun Hyung Jeong[a,b], Min Ho Park[a,b], Hyoun Ji Ha[a,b], Seong Jae Kang[a,b], Seong Jun Kang[a,b,*]

[a] Department of Advanced Materials Engineering for Information and Electronics, Kyung Hee University, Yongin 17104, Republic of Korea

[b] Integrated Education Program for Frontier Materials (BK21 Four), Kyung Hee University, Yongin 17104, Republic of Korea

Index

Figure S1. Schematic illustration of H-PH1000 electrode fabrication

Figure S2. CA images with (a) 1-layer PH1000 (b) 2-layer PH1000 (c) 3-layer PH1000

Figure S3. Sheet resistance distribution of H-PH1000 layers

Figure S4. Operational lifetime measurements of 3-layer H-PH1000 and ITO QLEDs

Figure S5. Voltage-current density characteristics of QLEDs

Table S1. Characteristics comparison of ITO and alternative electrodes

Table S2. Summarized EL characteristics of QLEDs with alternatives to ITO electrodes

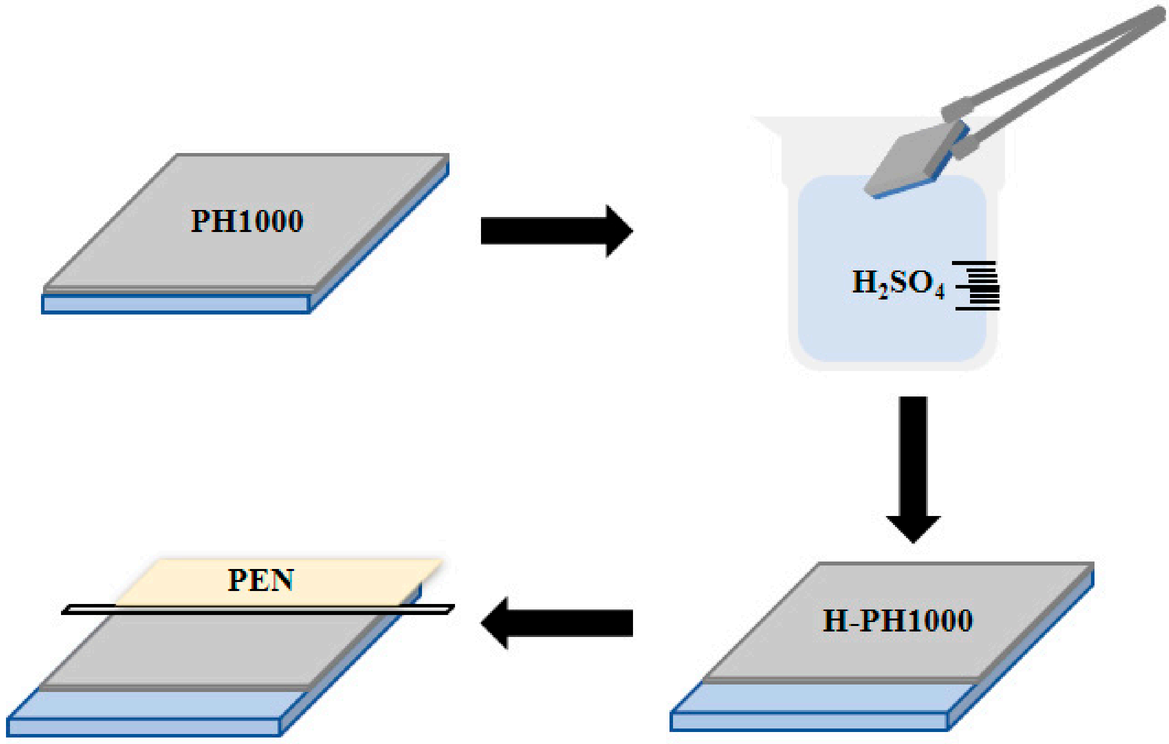

Figure S1. Schematic illustration of H-PH1000 electrode fabrication

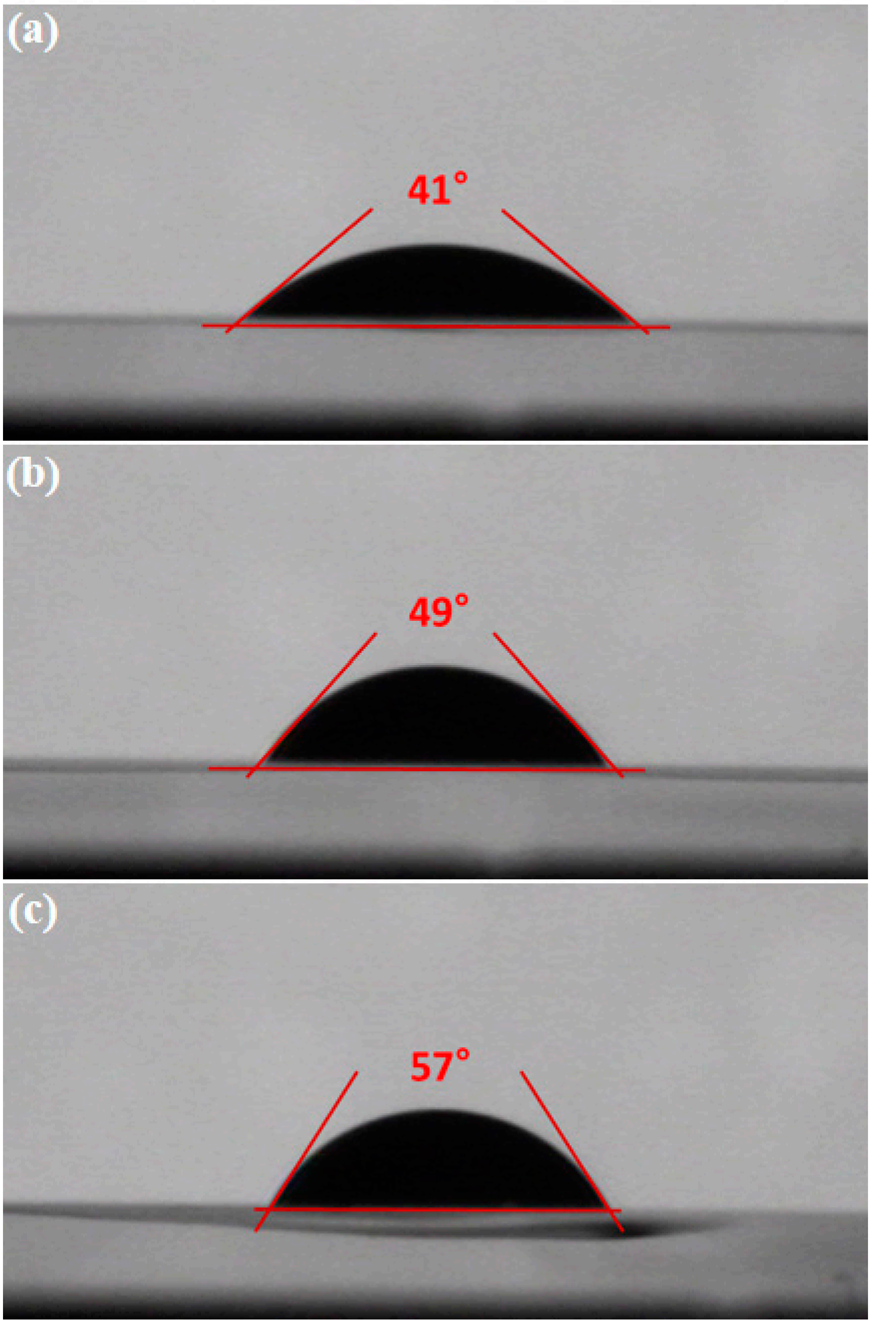

Figure S2. CA images with (a) 1-layer PH1000 (b) 2-layer PH1000 (c) 3-layer PH1000

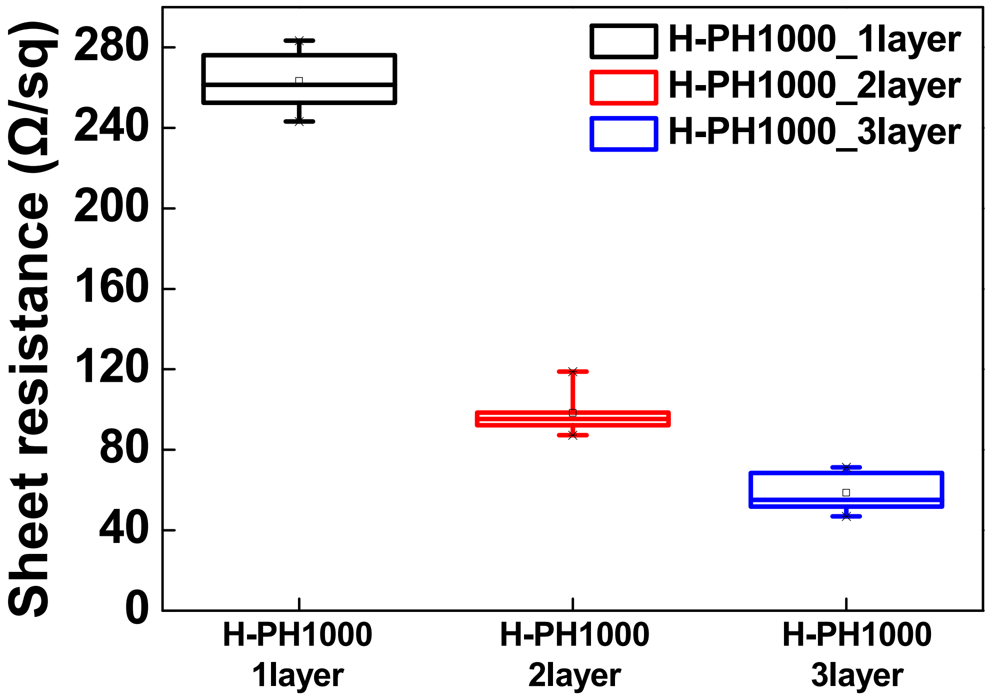

Figure S3. Sheet resistance distribution of H-PH1000 layers

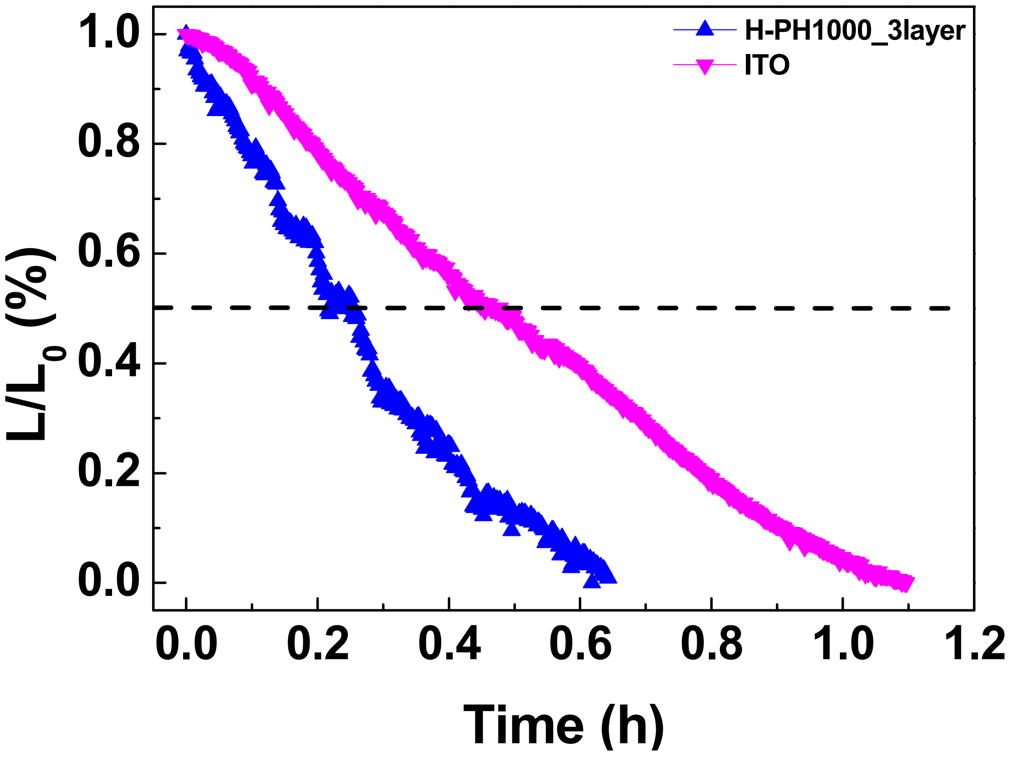

Figure S4. Operational lifetime measurements of 3-layer H-PH1000 and ITO QLEDs

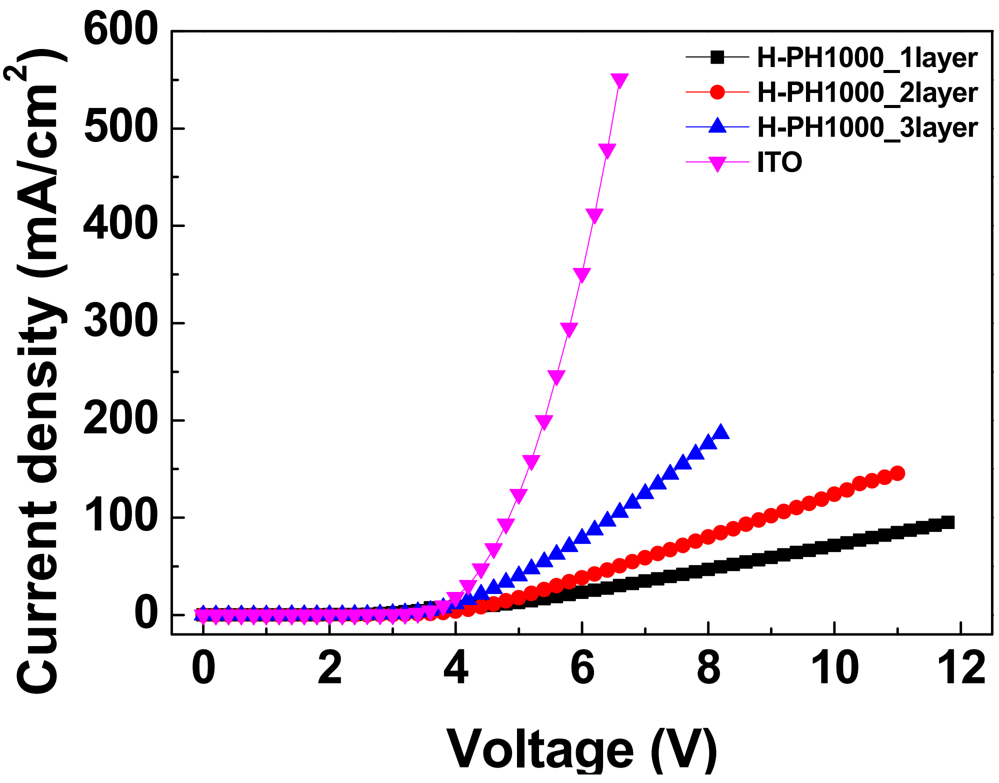

Figure S5. Voltage-current density characteristics of QLEDs

Materials	*Sheet resistance*	*Price*	*Transmittance*	*Fabrication method*	*Ref*
ITO	*10 ~ 30 Ω /sq*	*400 $ / m^2*	*Over 82 %*	*Sputtering*	*12*
PEDOT:PSS	*100 ~ 1000 Ω /sq*	*0.61 $ / ml*	*Over 80%*	*Solution*	*13*
Graphene	*100 ~ 300 Ω /sq*	*113 $ / $inch^2$*	*Over 90 %*	*Chemical vapor deposition*	*12*
CNT	*200 ~ 400 Ω /sq*	*250 $ / gram*	*Over 90 %*	*Chemical vapor deposition*	*12*
Silver nanowire	*10 – 30 Ω /sq*	*40 $ / m^2*	*Over 90 %*	*Solution*	*14, 15*

Table S1. Characteristics comparison of ITO and alternative electrodes

Electrodes	***Max. L (cd/m^2)***	***Max. CE (cd/A)***	***Max. EQE (%)***	***Color***	***Ref.***
3layer H-PH1000	46,663	46.53	11.01	Green	This work
ITO	68,977	15.62	3.69	Green	This work
Graphene	14,330	20.77	5.3	Green	40
Silver nanowire	27,310	45.99	N/A	Green	41
Carbon nano tube	N/A	N/A	N/A	IR	42

Table S2. Summarized EL characteristics of QLEDs with alternatives to ITO electrodes
